# Role of Trimetazidine in Ischemic Preconditioning in Patients With Symptomatic Coronary Artery Disease

**DOI:** 10.1097/MD.0000000000001161

**Published:** 2015-08-21

**Authors:** Leandro M.A. Costa, Paulo C. Rezende, Rosa M.R. Garcia, Augusto H. Uchida, Luis Fernando B.C. Seguro, Thiago L. Scudeler, Edimar A. Bocchi, Jose E. Krieger, Whady Hueb, José Antonio F. Ramires, Roberto Kalil Filho

**Affiliations:** From the Department of Atherosclerosis, Heart Institute (InCor) of the University of São Paulo, São Paulo, Brazil.

## Abstract

Ischemic preconditioning (IP) is a powerful cardioprotective cellular mechanism that has been related to the “warm-up phenomenon” or “walk-through” angina, and has been documented through the use of sequential exercise tests (ETs). It is known that several drugs, for example, cromokalim, pinacidil, adenosine, and nicorandil, can interfere with the cellular pathways of IP. The purpose of this article is to report the effect of the anti-ischemic agent trimetazidine (TMZ) on IP in symptomatic coronary artery disease (CAD) patients.

We conducted a prospective study evaluating IP by the analysis of ischemic parameters in 2 sequential ETs. In phase I, without TMZ, patients underwent ET1 and ET2 with a 30-minute interval between them. In phase II, after 1 week of TMZ 35 mg twice daily, all patients underwent 2 consecutive ETs (ET3 and ET4). IP was considered present when the time to 1.0-mm segment ST on electrocardiogram deviation (T-1.0 mm) and rate pressure product (RPP) were greater in the second of 2 tests. The improvement in T-1.0 mm and RPP were compared in the 2 phases: without TMZ and after 1-week TMZ to assess the action of such drug in myocardial protective mechanisms. ETs were analyzed by 2 independent cardiologists.

From 135 CAD patients screened, 96 met inclusion criteria and 62 completed the study protocol. Forty patients manifested IP by demonstrating an improvement in T-1.0 mm in ET2 compared with ET1, without the use of any drugs (phase I). In phase II, after 1-week TMZ, 26 patients (65%) did not show any incremental result in ischemic parameters in ET4 compared with ET3. Furthermore, of these patients, 8 (20%) had IP blockage.

In this study, TMZ did not add any benefit to IP in patients with stable symptomatic CAD.

## INTRODUCTION

Despite the progress of cardiology treatments in the last decades, cardiovascular diseases (CVD) remain the leading causes of death worldwide.^1^ Moreover, among CVD, ischemic heart disease represents the major cause of mortality.^1^ After the emergence of reperfusion therapies for the treatment of myocardial ischemic complications, many efforts have been put forth to discover any other cardioprotective mechanisms that could be translated into better clinical outcomes.^2^ One of this promising mechanisms is named Ischemic preconditioning (IP). IP is currently recognized as a powerful protective cellular mechanism in which brief and recurrent episodes of myocardial ischemia followed by reperfusion may self-protect one from prolonged ischemic injury and, thus, limit the size of myocardial infarction.^3^

In humans, IP may be assessed during coronary angioplasty,^4^ intermittent aortic cross-clamping during coronary artery bypass graft surgery,^5^ and during sequential exercise tests (ETs).^6–8^ The “warm-up phenomenon” or “walk-through” angina has been related to IP and documented in several studies in which sequential ETs were conducted.^9–12^

The improvement in ischemic parameters, such as the time to reach 1 mm of segment ST on electrocardiogram (ST) deviation and the time to develop angina in the second of 2 sequential tests, is considered a manifestation of IP. Studies that evaluated invasive parameters during sequential ETs demonstrated that the improvement in clinical and electrocardiographic parameters was confirmed by invasive measurements because myocardial lactate production and oxygen consumption were lower in the second of 2 sequential tests.^13,14^ Interestingly, this phenomenon occurred irrespective of coronary flow changes, which also confirms the intracellular mechanism of IP.^13,14^

The cellular mechanisms have been extensively studied, but these pathways remain controversial and are only partially understood.^15,16^ Although many pathways related to ischemic-reperfusion injury have recently been discovered and seem to be involved with cardioproctetive mechanisms,^17–18^ it has been proposed that endogenous opioids, adenosine, and bradykinin released during the brief ischemia of the IP protocol bind to specific G-protein coupled receptors (GPCR).^19–21^ Adenosine is released from myocytes as a consequence of adenosine triphosphate (ATP) breakdown during the preconditioning ischemia, and acts by switching on a complex intracellular signaling pathway that results ultimately in decreased ATP utilization. Liu et al^19^ showed that adenosine released during preconditioning occlusion (5 minutes of ischemia followed by 10 minutes of recovery) stimulates cardiac A1 receptors, which leaves the heart protected against infarction in the rabbit heart.

These GPCR stimulations (opioids, adenosine, and bradykinin) trigger different signaling pathways, including activation of phosphatidylinositol 3-kinase (PI3K), protein kinase B (Akt), and protein kinase C (PKC).^15,16^ In turn, it leads to the translocation of protein kinases from the cytoplasm to sarcolemma,^20^ where it phosphorylates a substrate protein, the ATP-sensitive K^+^ (KATP) channel.^22^ It has been proposed that the opening of KATP channels during IP is an important event because the cardioprotection can be abolished by treatment with KATP channel blockers such as glibenclamide and repaglinide,^23–25^ and mimicked by the channel openers such as cromokalim and pinacidil.^26^ Nicorandil, an antianginal drug used for the treatment of ischemic heart disease, is believed to have an intrinsic mechanism of selective activation of KATP channels at the sarcolemmal and mitochondrial level. Ahmed et al^27^ showed that nicorandil was effective in attenuating the ischemia/reperfusion-induced ventricular arrhythmias, creatinekinase-MB release, lactate accumulation, and oxidative stress in rats. In a study in elderly patients undergoing coronary angioplasty, the IP impairment was reversed by nicorandil administration.^28^

Trimetazidine (TMZ), another anti-ischemic agent recently introduced into clinical practice, acts by changing myocardial metabolism and exerts its antianginal effects regardless of hemodynamic changes. It acts by inhibiting selectively the enzyme 3-ketoacyl coenzyme A thiolase (3-KAT), which is responsible for oxidation of free fatty acids in the myocardium.^29^ This leads to a shift in myocardial metabolism from free fatty acid utilization to predominantly glucose metabolism,^29^ enhancing oxygen efficiency during myocardial ischemia. As a consequence, TMZ seems to reduce intracellular acidosis^30^ and the production of intracellular free radicals,^31^ protecting, in animal models, myocyte function.^32^

With the increased knowledge of some cellular pathways of IP, some medications have been proposed as potential pharmacologic preconditioning mimetics. In this way, despite its cardioprotective profile and the demonstration that TMZ may increase adenosine levels in humans,^33^ the results of studies on TMZ action in IP are controversial.^34,35^ The few studies that have addressed this question were conducted in animal models.^35^ Moreover, in humans, there are no studies that have evaluated TMZ effects in the expression of IP. Thus, the aim of the present study was to evaluate IP in patients with symptomatic coronary artery disease (CAD) and preserved ventricular function, and to assess whether the use of TMZ influenced this important mechanism of myocardial protection.

## MATERIALS AND METHODS

This study was a prospective, nonrandomized trial that received approval of the local ethics committee of the Heart Institute of the University of São Paulo and was conducted in accordance with the Declaration of Helsinki. All patients gave written informed consent to participate in the study.

All the patients included in this study had multivessel CAD (internal diameter reduction ≥70% of at least 2 major coronary branches), preserved left ventricular function, confirmed by transthoracic echodopplercardiography (left ventricular ejection fraction >0.45) and documentation of reproducible positive ETs for myocardial ischemia (horizontal or downsloping ST-segment depression ≥1.0 mm). All patients had normal hepatic and renal function. The exclusion criteria were left ventricular hypertrophy, myocardial infarction in the last 3 months, heart failure, cardiomyopathy, valvular disease, electrocardiogram changes, and conduction defects that could interfere with the interpretation of ST-segment changes. Furthermore, oral antihyperglycemic agents, calcium channel and beta-adrenergic blockers, angiotensin-converting enzyme inhibitors, angiotensin II receptor blockers, and caffeine were withdrawn 5 days before the study. Only nitrates were permitted, but they were withdrawn 12 hours before consecutive treadmill ETs.

The study protocol included 2 phases: after a “washout” period of 7 days with no cardiovascular medications or antihyperglycemic drugs, all patients underwent 2 consecutive treadmill ETs (ET1 and ET2), with an interval of 30 minutes between them to identify the ischemia and document the magnitude of IP by the difference in ischemic parameters between the 2 tests; all patients received only 35 mg twice daily of TMZ (VASTAREL MR) for 6 days. On the seventh day, the patients received 35 mg of TMZ for 60 minutes (time to peak plasma levels) before the beginning of the 2 consecutive ETs (ET3 and ET4). The time interval between ET3 and ET4 was similar to that in phase I. All tests were performed 1 hour after lunchtime.

### Treadmill Exercise Testing

All patients underwent computer-assisted treadmill ETs, symptom limited, according to the Bruce protocol, with a recovery phase of 6 minutes. The time interval between the consecutive tests was 30 minutes. We used an MAT2100 treadmill and a Fukuda Denshi ML8000 Stress Test system (Fukuda Denshi; Bunkyo-ku, Tokyo, Japan).

A 12-lead electrocardiogram, heart rate, and arterial blood pressure were obtained for all patients while in the standing position at baseline. A 12-lead electrocardiogram was also obtained at each 1.0-minute interval during exercise, at peak exercise, each minute up to 6 minutes after the exercise phase, at the onset of 1.0-mm ST-segment depression, at major ST-segment deviation, at the onset of angina pectoris, and when it was clinically relevant. The electrocardiogram was continuously monitored during the exercise and recovery phases, and an up-to-date averaged electrocardiographic signal of all leads was continuously displayed on the computer screen. The level of the ST-segment deviation was based on visual analyses of the 0.08 seconds after the J point by 2 independent cardiologists in a blind fashion. In case of disagreement, a third cardiologist was consulted and the matter was resolved by consensus. Only the horizontal or downsloping ST-segment depressions were considered for the time to onset of 1.0-mm ST-segment depression evaluation (T-1.0 mm). Criteria for interrupting the ET were ST-segment depression ≥3.0 mm, ST-segment elevation ≥2.0 mm, maximum age-related heart rate, severe chest pain, physical exhaustion, severe arterial hypotension, severe arterial hypertension, and complex or sustained arrhythmias, or both. The following parameters were systematically measured: resting heart rate and arterial blood pressure, heart rate and arterial blood pressure at peak exercise, T-1.0 mm in seconds, rate pressure product (RPP) at the onset of 1.0 mm ST-segment depression, and exercise duration in seconds.

### IP Analysis

The parameters assessed during sequential ETs to characterize the presence of IP were T-1.0 mm and RPP. The improvement in these ischemic parameters in ET2 compared with ET1 indicated IP.

### Statistical Analysis

Two-way ANOVA with repeated measures followed by the Bonferroni test were used to compare T-1.0 mm and RPP data. Comparisons of the remaining continuous or discrete variables between the 2 phases were performed using an unpaired Student *t* or χ^2^ test, respectively. Fisher test was used when appropriate. Data were expressed as means ± standard deviation and in the figures as median and interquartile ranges. A value of *P* < 0.05 was considered significant. The software SPSS version 20 was used for all statistical analysis.

## RESULTS

From 135 CAD patients followed at our tertiary hospital, 96 met inclusion criteria for this study. Of these patients, 62 completed the study protocol, and 40 manifested IP by demonstrating improvement in the time to reach 1.0 mm ST deviation in the second of 2 sequential ETs. These patients all underwent a second phase of sequential ETs after 1 week of TMZ.

The main demographic, biochemical, and clinical characteristics of these 40 patients are shown in Table 1.

**TABLE 1:**
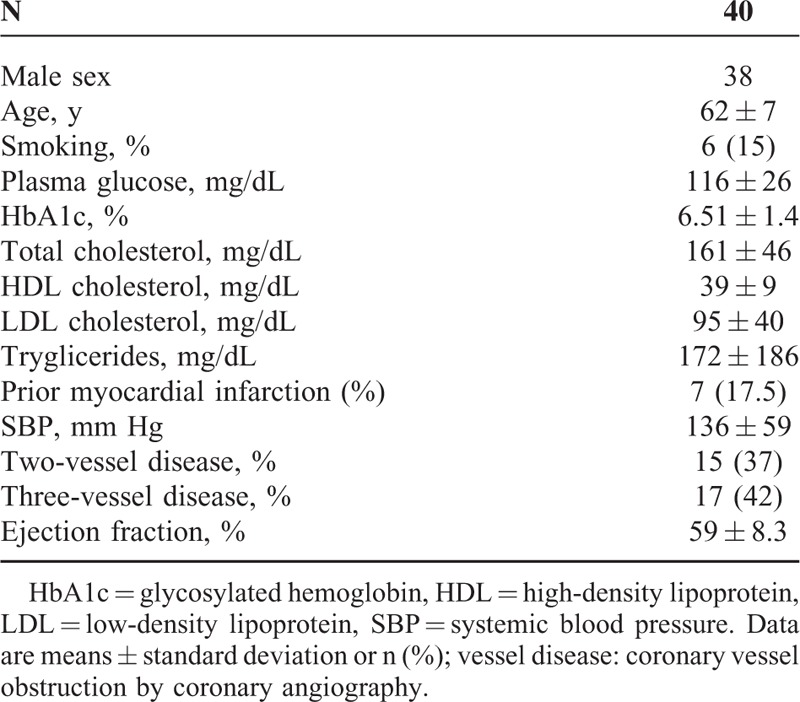
The Main Demographic, Biochemical, and Clinical Characteristics of the Study Population

### Results of Treadmill Exercise Tests

All 40 patients achieved 1.0 mm ST-segment depression during the ETs (ET1 and ET2).

### Phase I—Without TMZ

During phase I, all patients demonstrated improvement in the T-1.0 mm in ET2 compared with ET1, demonstrating the expression of IP by this parameter. RPP suggested improvement, but the results were not statistically significant (Table 2).

**TABLE 2:**
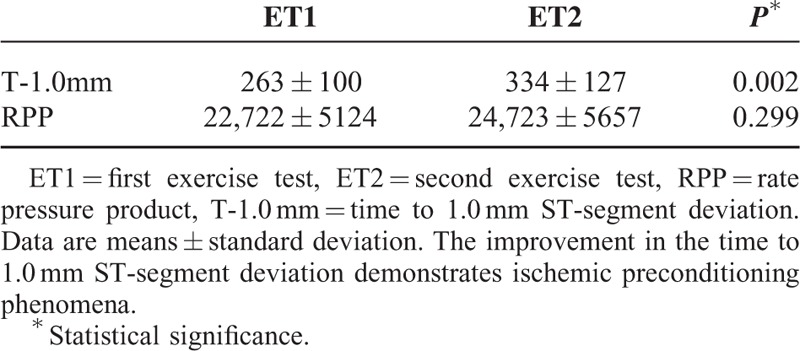
Time to 1.0 mm ST-Segment Deviation and Rate Pressure Product of 40 Study Patients in the First Phase Without Trimetazidine

### Phase II—With TMZ

**FIGURE 1:**
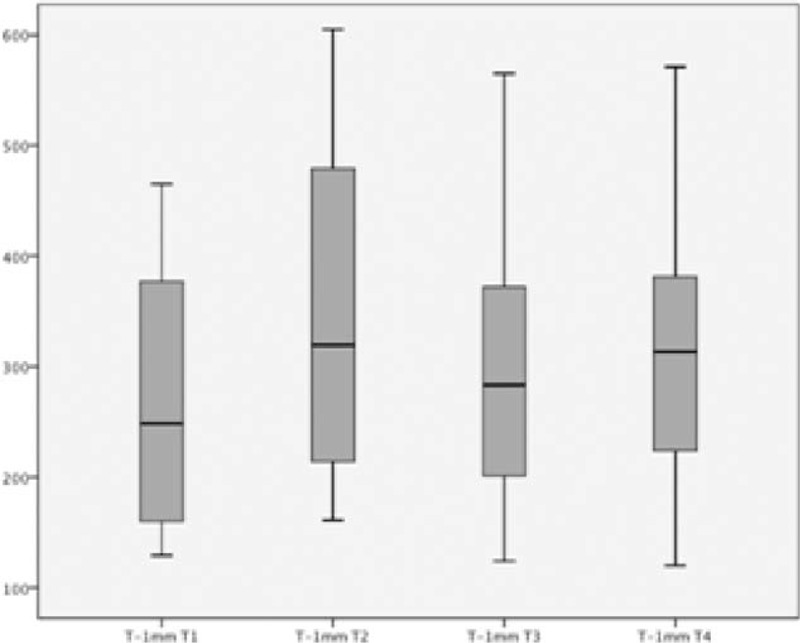
Time to 1.0 mm ST-segment deviation in the 4 exercise tests. The first 2 tests were performed without the use of medications, and the last 2 tests (T3 and T4) were performed after 1 week of TMZ. The whiskers represent minimum and maximum data, and the middle lines represent median and interquartile range.

After 6 days of TMZ 35 mg twice daily, the 40 patients underwent 2 sequential ETs (ET3 and ET4). The results of the 4 tests of the 2 phases are shown in Figure 1.

### Analysis of Variation of T-1.0 mm and RPP

**FIGURE 2:**
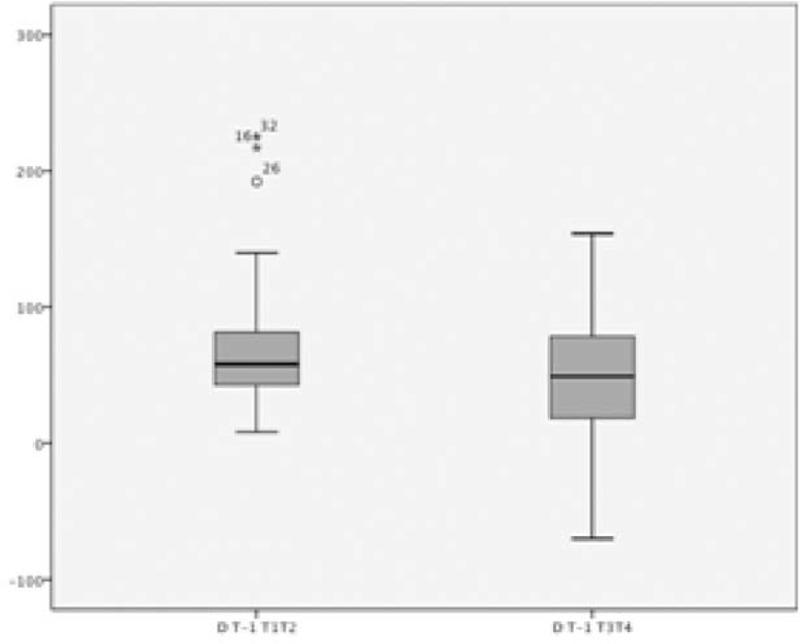
Results of Delta 1 and Delta 2 of T-1.0 mm. Delta 1: variation between values of ET1 and ET2; Delta2: variation between values of ET3 and ET4. The whiskers represent minimum and maximum data, and the middle lines represent median and interquartile range. The stars and the small circle represent the outliers.

The variation of the 2 parameters, T-1.0 mm and RPP, was compared between ET1 and ET2 in phase I (Delta1), and ET3 and ET4 in phase II (Delta 2), and the difference between them (Difference Delta). In the entire group, the comparison between Delta 1 and Delta 2 of T-1.0 mm was 71 ± 49 and 48 ± 47 (*P* = 0.009), respectively, and the Difference Delta −23 ± 53. This demonstrates a lack of power of IP after 1 week of TMZ (Figure 2). The comparison between Delta 1 and Delta 2 of RPP was 2000 ± 1949 and 1395 ± 2006 (*P* = 0.069), respectively, with the difference of Delta of −605 ± 2050.

From the initial 40 patients, the improvement in T-1.0 mm observed in the first phase was not incremental in 26 patients (65%) (Negative Delta = Delta −) by analysis of T-1.0 mm (Table 3). Moreover, of these 26 patients, 8 (20%) showed the blockage of IP, whereas 18 (45%) did not show any incremental result in terms of T-1.0 mm difference, as is shown in Table 2.

**TABLE 3:**
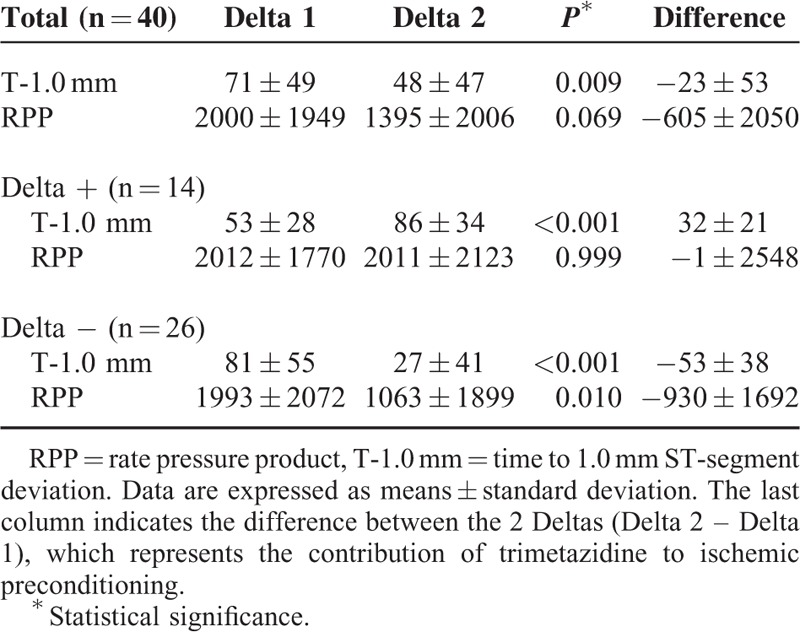
The Expression of Ischemic Preconditioning by the Improvement in the Time to 1.0 mm ST-Segment Deviation and Rate Pressure Product in the First Phase (Delta 1) and in the Second Phase (Delta 2)

On the contrary, 14 patients (35%) reached an incremental result (Positive Delta = Delta +), as the Delta T-1.0 mm in the second phase with TMZ was higher than that observed in the first phase (Delta 2 = 86 ± 34 and Delta 1 = 53 ± 28, *P* < 0.001). However, in this group, there were no incremental changes in RPP (Delta 1 = 2012 ± 1770, Delta 2 = 2011 ± 2123, Difference Delta = −1 ± 2548, *P* = 0.999).

The results of each ET in terms of T-1.0 mm and RPP in the 2 groups (Delta + and Delta −) are shown in Table 4.

**TABLE 4:**
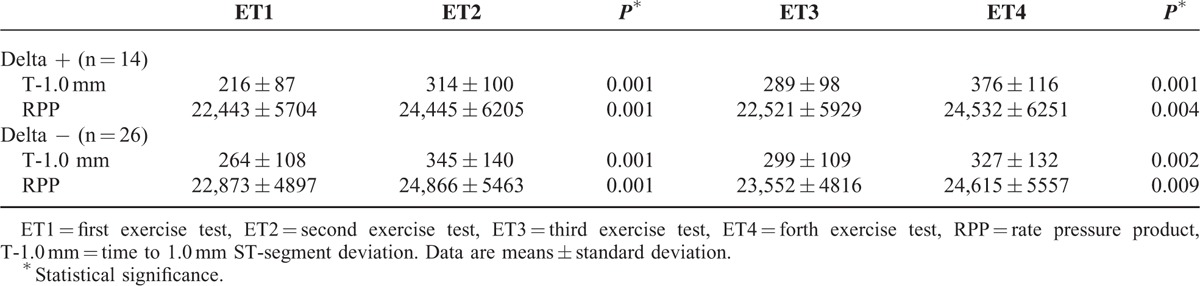
Time to 1.0 mm ST-Segment Deviation and Rate Pressure product in the 2 Phases (Phase I: Exercise Tests 1 and 2; Phase II: Exercise tests 3 and 4) in the 2 Groups of Patients (Delta + and Delta −)

## DISCUSSION

Although TMZ has been extensively studied in ischemic situations, this study addressed a novel finding on the action of this pharmacological agent in a cardioprotective mechanism. We report for the first time in humans, the effects of TMZ on the warm-up phenomenon, which is considered a clinical model of IP.^11^

The findings of the present study show that in the first phase, in the absence of TMZ, ischemia observed in the second ET was significantly attenuated compared with ischemia in the first exercise. The consistent attenuation of electrocardiographic signs of ischemia can be observed by the improvement in the tolerance to myocardial ischemia and ischemic threshold.^6,9–11,13,36^

Although all patients achieved improvement in T-1.0 mm and RPP consistent with the expression of IP in the phase without any drugs, we did not observe any additional improvement in ischemic parameters with TMZ for 7 days, in phase II. Indeed, these parameters were lower with TMZ use compared with the phase without any drugs. In addition, in a significant percentage of patients who demonstrated IP in the first phase, IP was blocked in the second phase with TMZ use.

The findings of the present study are similar to those of Minners et al^34^ in an experimental study with isolated rat hearts. They showed that TMZ reversed the cardioprotection afforded by other medications and also by IP. In addition, they hypothesized that alterations in mitochondrial homeostasis may make it less adaptive after an ischemic insult. Interestingly, these authors showed that some drugs that induce mitochondrial stress, such as dinitrophenol and cyclosporine A, can trigger preconditioning, and drugs initially considered as mitochondrial protectives may limit preconditioning-like cardioprotection. They postulate that this action of TMZ may be partially explained by its limiting of preconditioning-induced cardioprotection.

The complex cellular cascades underlying IP seem to converge at the opening of the mitochondrial KATP channel, which leads to the influx of potassium into mitochondria. In turn, it causes modifications of the inner membrane and increases mitochondrial volume and consequently the activity of the electron transport chain, resulting in mitochondrial “swelling” and in increased ATP production. It appears that some agents or situations that cause mitochondria stress and subsequent swelling could activate mechanisms that render the mitochondria more resistant to a subsequent insult. Thus, preventing mitochondria swelling with TMZ may attenuate cardioprotective mechanisms.^34^

Moreover, ATP has an inhibitory effect on the sarcolemma KATP channel, and in a situation of ATP shortage, like during ischemia, it may induce the opening of such channels. It is accepted that TMZ leads to changes in myocardial metabolism, shifting the utilization of free fatty acids into glucose metabolism, and hence it leads to higher ATP production in the myocardium. Thereby, the rise in ATP production may also be responsible for KATP channel inhibition and IP prevention.

On the contrary, Kara et al^35^ suggested in a rat model that TMZ preserved IP and pharmacological preconditioning. In this study, the cardioprotection afforded by ischemia and by carbachol in terms of ventricular arrhythmias and the size of myocardial infarction were preserved by TMZ. Differently from Minners’ study with isolated rat hearts, Kara studied anesthetized rats and evaluated different parameters. These differences may partially explain the opposite results of Kara's and Minners’ studies.

Many factors may influence outcomes in different ways in CAD patients,^37^ and the actions of drugs in myocardial responses may also play distinct roles in IP. Thus, despite the antianginal effects of TMZ have been addressed in some studies, its effects on IP are still not clearly known. Moreover, ischemia and IP are 2 different situations in the myocardium, which deserve to be clearly differentiated. Myocardial ischemia must be avoided, and antianginal drugs act in this way. However, once ischemia has occurred, it may trigger a preconditioning cellular program. In this scenario, some drugs may block this phenomenon, whereas others may not interfere or may theoretically stimulate it. Thus, some drugs, such as glibenclamide^24,38,39^ or repaglinide,^25^ have the potential to block IP. On the contrary, vildagliptin seems not to interfere with IP.^40^ Interestingly, antianginal drugs may not induce IP,^41^ and thereby these agents do not seem to be pharmacological preconditioning mimetics. Thus, such relevant information from this study suggests that TMZ may not be used for this purpose.

Although the analysis of IP by sequential ETs is a matter of debate among some authors, some invasive studies confirm that the improvement in electrocardiographic parameters is confirmed by the clinical assessment of angina and by invasive measurements such as myocardial lactate production. Thus, this methodology is one of the few noninvasive ways to measure human IP.

Another important consideration from our study is that once preconditioning is stimulated by the first ischemic insult, its powerful protective mechanism may not permit TMZ to confer any further additional protection. In addition, the chronic effect of TMZ was not assessed in the present study.

In summary, our results reveal that TMZ does not improve IP in patients with stable multivessel CAD.

## CONCLUSION

In this study, TMZ did not have any benefit on IP in patients with symptomatic CAD.

## References

[1] Santulli G Epidemiology of cardiovascular disease in the 21st century: updated numbers and updated facts. *J Cardiovasc Dis* 2013; 1:1–2.

[2] Hausenloy DJ Yellon DM Myocardial ischemia-reperfusion injury: a neglected therapeutic target. *J Clin Invest* 2013; 123:92–100. 23281415 10.1172/JCI62874 PMC3533275

[3] Murry CE Jennings RB Reimer KA Preconditioning with ischemia: a delay of lethal cell injury in ischemic myocardium. *Circulation* 1986; 74:1124–1136. 3769170 10.1161/01.cir.74.5.1124

[4] Deutsch E Berger M Kussmaul WG Adaptation to ischemia during percutaneous transluminal coronary angioplasty. Clinical, hemodynamic, and metabolic features. *Circulation* 1990; 82:2044–2051. 2242528 10.1161/01.cir.82.6.2044

[5] Yellon DM Alkhulaifi AM Parsleys WB Preconditioning the human myocardium. *Lancet* 1993; 342:276–277. 8101304 10.1016/0140-6736(93)91819-8

[6] Waters DD McCans JL Crean PA Serial exercise testing in patients with effort angina: variable tolerance, fixed threshold. *J Am Coll Cardiol* 1985; 6:1011–1015. 4045025 10.1016/s0735-1097(85)80302-8

[7] MacAlpin RN Kattus AA Adaption to exercise in angina pectoris. The electrocardiogram during treadmill walking and coronary angiographic findings. *Circulation* 1966; 33:183–201. 25823091 10.1161/01.cir.33.2.183

[8] Ylitalo K Jama L Raatikainen P Adaptation to myocardial ischemia during repeated dynamic exercise in relation to findings at cardiac catheterization. *Am Heart J* 1996; 131:689–697. 8721640 10.1016/s0002-8703(96)90272-0

[9] Jaffe MD Quinn NK Warm-up phenomenon in angina pectoris. *Lancet* 1980; 2:934–936. 6107586 10.1016/s0140-6736(80)92101-7

[10] Stewart RA Simmonds MB Williams MJ Time course of “warm-up” in stable angina. *Am J Cardiol* 1995; 76:70–73. 7793408 10.1016/s0002-9149(99)80804-2

[11] Maybaum S Ilan M Mogilevsky J Improvement in ischemic parameters during repeated exercise testing: a possible model for myocardial preconditioning. *Am J Cardiol* 1996; 78:1087–1091. 8914868 10.1016/s0002-9149(96)90057-0

[12] Tomai F Warm up phenomenon and preconditioning in clinical practice. *Heart* 2002; 87:99–100. 11796535 10.1136/heart.87.2.99 PMC1766987

[13] Okazaki Y Kodama K Sato H Attenuation of increased regional myocardial oxygen consumption during exercise as a major cause of warm-up phenomenon. *J Am Coll Cardiol* 1993; 21:1597–1604. 8496525 10.1016/0735-1097(93)90374-a

[14] Williams DO Bass TA Gewirtz H Adaptation to the stress of tachycardia in patients with coronary artery disease: insight into the mechanism of the warm-up phenomenon. *Circulation* 1985; 71:687–692. 3971538 10.1161/01.cir.71.4.687

[15] Downey JM Davis AM Cohen MV Signaling pathways in ischemic preconditioning. *Heart Fail Rev* 2007; 12:181–188. 17516169 10.1007/s10741-007-9025-2

[16] Hausenloy DJ Yellon DM Preconditioning and postconditioning: united at reperfusion. *Pharmacol Ther* 2007; 116:173–191. 17681609 10.1016/j.pharmthera.2007.06.005

[17] Paulis L Fauconnier J Cazorla O Activation of Sonic hedgehog signaling in ventricular cardiomyocytes exerts cardioprotection against ischemia reperfusion injuries. *Sci Rep* 2015; 5:7983. 25613906 10.1038/srep07983 PMC4303926

[18] Qi D Atsina K Qu L The vestigial enzyme D-dopachrome tautomerase protects the heart against ischemic injury. *J Clin Invest* 2014; 124:3540–3550. 24983315 10.1172/JCI73061 PMC4109524

[19] Liu GS Thornton J Van Winkle DM Protection against infarction afforded by preconditioning is mediated by adenosine Al receptors in rabbit heart. *Circulation* 1991; 84:350–356. 2060105 10.1161/01.cir.84.1.350

[20] Wall TM Sheehy R Hartman JC Role of bradykinin in myocardial preconditioning. *J Pharmacol Exp Ther* 1994; 270:681–689. 8071859

[21] Schultz JEJ Rose E Yao Z Evidence for involvement of opioid receptors in ischemic preconditioning in rat hearts. *Am J Physiol* 1995; 268:H2157–H2161. 7771566 10.1152/ajpheart.1995.268.5.H2157

[22] Ytrehus K Liu Y Downey JM Preconditioning protects ischemic rabbit heart by protein kinase C activation. *Am J Physiol* 1994; 266:H1145–H1152. 8160817 10.1152/ajpheart.1994.266.3.H1145

[23] Cleveland JC Meldrum DR Cain BS Oral sulfonylurea hypoglycemic agents prevent ischemic preconditioning in human myocardium: two paradoxes revisited. *Circulation* 1997; 96:29–32. 9236412 10.1161/01.cir.96.1.29

[24] Tomai F Crea F Gaspardone A Ischemic preconditioning during coronary angioplasty is prevented by glibenclamide, a selective ATP-sensitive K+ channel blocker. *Circulation* 1994; 90:700–705. 8044938 10.1161/01.cir.90.2.700

[25] Hueb W Uchida AH Gersh BJ Effect of a hypoglycemic agent on ischemic preconditioning in patients with type 2 diabetes and stable angina pectoris. *Coron Artery Dis* 2007; 18:55–59. 17172931 10.1097/MCA.0b013e328011c0a9

[26] Grover GJ Dzwonczyk SS Parham CS The protective effects of cromokalim and pinacidil on reperfusion function and infarct size in isolated perfused rat hearts and anaesthetised dogs. *Cardiovasc Res* 1992; 26:1054–1062. 2285630 10.1007/BF01857755

[27] Ahmed LA Salem HA Attia AS Pharmacological preconditioning with nicorandil and pioglitazone attenuates myocardial ischemia/reperfusion injury in rats. *Eur J Pharmacol* 2011; 663:51–58. 21549700 10.1016/j.ejphar.2011.04.038

[28] Lee TM Su SF Chou TF Loss of preconditioning by attenuated activation of myocardial ATP-sensitive potassium channels in elderly patients undergoing coronary angioplasty. *Circulation* 2002; 105:334–340. 11804989 10.1161/hc0302.102572

[29] Kantor PF Lucien A Zozak R The antianginal drug trimetazidine shifts cardiac energy metabolism from fatty acid oxidation to glucose oxidation by inhibiting mitochondrial long-chain 3-ketoacyl coenzyme A thiolase. *Circ Res* 2000; 86:580–588. 10720420 10.1161/01.res.86.5.580

[30] El Banani H Bernard M Baetz D Changes in intracellular sodium and pH during ischaemia-reperfusion are attenuated by trimetazide. Comparison between low- and zero-flow ischaemia. *Cardiovasc Res* 2000; 47:688–696. 10974217 10.1016/s0008-6363(00)00136-x

[31] Maupoil V Rochette L Tabard A Direct measurement of free radical generation in isolated rat heart by electron paramagnetic resonance spectroscopy: effect of trimetazidine. *Adv Exp Med Biol* 1990; 264:373–376. 2173876 10.1007/978-1-4684-5730-8_58

[32] Fantini E Demaison L Sentex E Some biochemical aspects of the protective effect of trimetazidine on rat cardiomyocytes during hypoxia and reoxygenation. *J Mol Cell Cardiol* 1994; 26:949–958. 7799450 10.1006/jmcc.1994.1116

[33] Blardi P De Lalla A Volpi L Increase of adenosine plasma levels after oral trimetazidine: a pharmacological preconditioning? *Pharmacol Res* 2002; 45:69–72. 11820865 10.1006/phrs.2001.0905

[34] Minners J van den Bos EJ Yellon DM Dinitrophenol, cyclosporine A, and trimetazidine modulate preconditioning in the isolated rat heart: support for a mitochondrial role in cardioprotection. *Cardiovasc Res* 2000; 47:68–73. 10869531 10.1016/s0008-6363(00)00069-9

[35] Kara AF Demiryurek S Celik A Effects of trimetazidine on myocardial preconditioning. *Eur J Pharmacol* 2004; 503:135–145. 15496308 10.1016/j.ejphar.2004.09.037

[36] Tomai F Crea F Danesi A Mechanisms of the warm-up phenomenon. *Eur Heart J* 1996; 17:1022–1027. 8809519 10.1093/oxfordjournals.eurheartj.a014997

[37] Santulli G Coronary heart disease risk factors and mortality. *JAMA* 2012; 307:1137. 22436947 10.1001/jama.2012.323

[38] Gross GJ Auchampach JA Blockade of ATP-sensitive potassium channels prevents myocardial preconditioning in dogs. *Circ Res* 1992; 70:223–233. 1310443 10.1161/01.res.70.2.223

[39] Ferreira BM Moffa PJ Falcão A The effects of glibenclamide, a K(ATP) channel blocker, on the warm-up phenomenon. *Ann Noninvasive Electrocardiol* 2005; 10:356–362. 16029388 10.1111/j.1542-474X.2005.00650.x PMC6932067

[40] Rahmi RM Uchida AH Rezende PC Effect of hypoglycemic agents on ischemic preconditioning in patients with type 2 diabetes and symptomatic coronary artery disease. *Diabetes Care* 2013; 36:1654–1659. 23250803 10.2337/dc12-1495 PMC3661846

[41] Opie LH Preconditioning and metabolic anti-ischaemic agents. *Eur Heart J* 2003; 24:1854–1856. 14563345 10.1016/s0195-668x(03)00439-1

